# Mutations in the *EPHA2* Gene Are a Major Contributor to Inherited Cataracts in South-Eastern Australia

**DOI:** 10.1371/journal.pone.0072518

**Published:** 2013-08-27

**Authors:** Alpana Dave, Kate Laurie, Sandra E. Staffieri, Deepa Taranath, David A. Mackey, Paul Mitchell, Jie Jin Wang, Jamie E. Craig, Kathryn P. Burdon, Shiwani Sharma

**Affiliations:** 1 Department of Ophthalmology, Flinders University, Bedford Park, SA, Australia; 2 Centre for Eye Research Australia, University of Melbourne, Royal Victorian Eye and Ear Hospital, Melbourne, Australia; 3 Royal Children’s Hospital, Melbourne, Australia; 4 Lions Eye Institute, University of Western Australia, Centre for Ophthalmology and Visual Science, Perth, Australia; 5 Discipline of Medicine, University of Tasmania, Hobart, Australia; 6 Centre for Vision Research, Department of Ophthalmology and Westmead Millennium Institute, The University of Sydney, Sydney, NSW, Australia; University of Valencia, Spain

## Abstract

Congenital cataract is the most common cause of treatable visual impairment in children worldwide. Mutations in many different genes lead to congenital cataract. Recently, mutations in the receptor tyrosine kinase gene, *EPHA2,* have been found to cause congenital cataract in six different families. Although these findings have established *EPHA2* as a causative gene, the total contribution of mutations in this gene to congenital cataract is unknown. In this study, for the first time, a population-based approach was used to investigate the frequency of disease causing mutations in the *EPHA2* gene in inherited cataract cases in South-Eastern Australia. A cohort of 84 familial congenital or juvenile cataract index cases was screened for mutations in the *EPHA2* gene by direct sequencing. Novel changes were assessed for segregation with the disease within the family and in unrelated controls**.** Microsatellite marker analysis was performed to establish any relationship between families carrying the same mutation. We report a novel congenital cataract causing mutation c.1751C>T in the *EPHA2* gene and the previously reported splice mutation c.2826-9G>A in two new families. Additionally, we report a rare variant rs139787163 potentially associated with increased susceptibility to cataract. Thus mutations in *EPHA2* account for 4.7% of inherited cataract cases in South-Eastern Australia. Interestingly, the identified rare variant provides a link between congenital and age-related cataract.

## Introduction

Cataract is an opacification of the crystalline lens. Congenital and juvenile cataract form a disease spectrum with presentation from birth or during early childhood and are generally referred to as congenital cataract [1]. Congenital cataract is the leading cause of childhood blindness and accounts for 1–6 and 5–15 cases per 10,000 live births respectively, in developed and poor regions of developing countries [2], [3]. Inherited congenital cataract accounts for one quarter of the cases [4]. Its genetic heterogeneity is evidenced by the presence of causative mutations in at least 24 structurally and functionally important genes in the lens, with mutations in ten crystallin genes accounting for 50% of the known mutations [4]. Mutations in the *EPHA2* gene have been recently identified to cause congenital cataract [5], [6], [7], [8]. Five different causative mutations with autosomal dominant mode of inheritance have been reported each in an American, Australian, British and two Chinese families [5], [6], [8]. One mutation with autosomal recessive mode of inheritance was reported in a Pakistani family [7]. Age-related cataract, the major cause of blindness in the elderly, is believed to result from both genetic predisposition and environmental factors [9]. Synonymous and non-synonymous variants in the *EPHA2* gene have been associated with age-related cataract in multiple populations [5], [10], [11], [12]. Thus *EPHA2* has been implicated in both congenital and age-related cataract suggesting a vital role of this gene in lens development and in maintaining lens cell homeostasis and transparency.

To date, the overall genetic contribution of the *EPHA2* gene to inherited congenital cataract is not known. With this objective, in the present study, we screened a South-Eastern Australian cohort of familial cataract cases for causative mutations in the *EPHA2* gene. We report a novel causative missense mutation in the gene in one family and a previously reported splice mutation c.2826-9G>A [6] in two new families. Additionally, we report a rare non-synonymous variant in the gene that may be increasing susceptibility to cataract and, providing a link between congenital and age-related cataract.

## Materials and Methods

### Ethics Statement

Ethics approval for the study was obtained from the Southern Adelaide Clinical Human Research Ethics Committee, Adelaide, South Australia, the Royal Victorian Eye and Ear Hospital (RVEEH) Human Research Ethics Committee, Melbourne, Victoria, and The University of Sydney and Sydney West Area Human Research Ethics Committees, New South Wales, Australia. All participants gave written informed consent. Where participants were minor or unable to personally provide consent, written informed consent was obtained from the parent, legal guardian or an authorized person.

### Patient Recruitment

This study attempted to recruit all familial congenital and juvenile cataract cases in the last 12 years from three states of South-Eastern Australia, South Australia, Victoria and Tasmania, and thus approximates a population-based approach in this region. Probands with familial cataract and their family members were recruited from the Flinders Medical Centre (Adelaide), Women’s and Children’s Hospital (Adelaide), Royal Children’s Hospital (Melbourne) and RVEEH (Melbourne). RVEEH also served as a tertiary referral centre for patient recruitment from Tasmania. Family history of the disease in all the families was available through previous clinical records. Genomic DNA of recruited individuals was extracted from either whole blood or saliva or buccal swab using standard methods.

### Sequencing Analysis

Probands from 84 families were included in this study. All 17 exons of the *EPHA2* gene were amplified by PCR and sequenced at the Australian Genomic Research Facility (AGRF, Brisbane, Australia) or in-house by direct sequencing using BigDye Terminators (Applied Biosystems, Melbourne, Australia) according to the standard protocols. Primers used for sequencing are provided in Tables S1 and S2, respectively. Sequence of *EPHA2* in the probands was compared with the reference sequence (GenBank accession NM_004431.3) using Sequencher 4.1.0 (Gene Codes Corporation, Michigan, USA). Novel or potentially disease causing variants in an individual were assessed for segregation with the disease by direct sequencing in other available affected and unaffected family members. The control cohort comprised 270 elderly individuals, with no record of congenital cataract surgery, recruited from around Adelaide, South Australia, Australia. Out of these, 66 individuals had age-related cataract. Variant c.1751C>T in Exon 10 was screened in all the controls using a custom Taqman assay (Applied Biosystems) on a StepOne Plus RealTime PCR System (Applied Biosystems). The variant c.2875G>A in Exon 17 was screened in 218 out of 270 controls, due to failure of the assay in rest of the controls, using SNaPshot assay (Applied Biosystems) according to the manufacturer’s protocols using a probe with sequence- 5′-TCC TTG AGT CCC AGC AGG CTG TAG G- 3′. Out of the 218 screened controls, 58 individuals had age-related cataract. Additionally, participants from the Blue Mountains Eye Study (BMES) cohort [13] were screened for this variant. Briefly, the BMES study recruited individuals 50 years or older, from the Blue Mountains region, west of Sydney, to investigate ocular diseases in the population. From this cohort, 150 age-related cataract cases, with unilateral or bilateral cortical cataract, and 271 controls, with no cataract of any nature, were analyzed for the variant as described above.

### Haplotype Analysis

Haplotype analysis was performed, in the family previously reported to carry the splice mutation c.2826-9G>A (Family 16) [6] and the two new families (Family 42 and Family 83) found to carry the same mutation in this study. Four microsatellite markers namely D1S228, D1S507, D1S436 and D1S2644 in the telomeric region of chromosome 1p were typed in affected and unaffected family members as previously described [14]. Sequences of primers used for this analysis are provided in Table S3.

### Bioinformatics Analysis

Multiple sequence alignment of EPHA2 protein sequences from human (GenPept accession NP_004422.2), mouse (GenPept accession NP_034269.2), aquatic frog (GenPept accession AAH75556.1), rhesus monkey (GenPept accession NP_001035768.1), chicken (GenPept accession XP_001234814.2) and zebrafish (GenPept accession NP_571490.1) was performed using CLUSTALW [15]. Pathogenicity of the novel mutations or variants was predicted using Sorting Intolerant from Tolerant (SIFT) and Polymorphism phenotyping v2 (PolyPhen2) online tools [16], [17].

## Results and Discussion

Previous genetic studies have firmly established involvement of the *EPHA2* gene in development of congenital and age-related cataract [5], [6], [7], [8], [10], [11], [12]. The *EPHA2* gene encodes a 976 amino acid transmembrane tyrosine kinase receptor. The EPHA2 protein has an extracellular region containing the ligand-binding domain, cysteine-rich domain and two fibronectin domains, a transmembrane segment, and an intracellular region consisting of a juxtamembrane domain, tyrosine kinase domain, sterile alpha motif (SAM) and PDZ domain [18]. EPHA2 interacts with its ligands, ephrin-A1 or ephrin-A5, to bring about bidirectional signalling. This signalling plays an important role in cell-adhesion and cell-repulsion in a context dependent manner [19]. Disruption of this signalling in the lens is likely the underlying cause of cataract development. EPHA2 is expressed in human and mouse lens and co-localises with junctional proteins in lens fibre cells [10], [20]. The *Epha2* and *ephrin-A5* knockout mice develop progressive cataracts thus demonstrating the importance of EPHA2 signalling in maintaining transparency and architecture of the mammalian lens [10], [20], [21].

In this study, we aimed to determine genetic contribution of the *EPHA2* gene to inherited congenital cataract in an Australian cohort. Eighty-four probands with familial congenital or juvenile cataract, displaying either autosomal dominant or autosomal recessive mode of inheritance of the disease, were recruited from South Australia, Victoria and Tasmania. All 17 exons of the *EPHA2* gene were screened for mutations in the probands.

We identified two novel non-synonymous variants, c.493G>A in probands from Families 41 and 75 and c.2837G>A in the proband from Family 111. These variants did not segregate with the disease in these families suggesting that they were not disease causing changes. They were subsequently listed in dbSNP under IDs rs150790360 and rs149692543, respectively, following identification in a large scale exome sequencing project [22]. Furthermore, we identified three potential congenital cataract causing mutations in Exon 10, Intron 16 and Exon 17 of the *EPHA2* gene segregating with the disease in one or more families.

**Figure 1:**
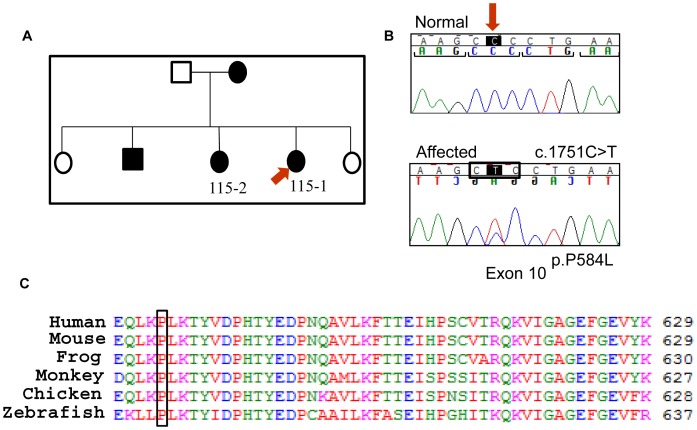
The causative mutation in Family 115. a) Pedigree of the Family 115. Solid boxes and circles indicate affected individuals. The individuals from whom DNA was available have been given a number. The proband is marked with a red arrow. b) Sequence chromatogram showing the mutation c.1751C>T, marked with an arrow, present in the proband (bottom). The mutation leads to change in a codon, marked by a box, substituting a proline by a leucine at position 584 in the protein. The translation reading frame is marked with brackets in the normal sequence (top). c) ClustalW alignment of the region of the human EPHA2 protein including residue 584 showing conservation of the mutated residue across species.

In Family 115, proband 115-1 presented with bilateral nuclear lens opacity. A novel mutation, c.1751C>T in Exon 10 of the gene, was observed (Figure 1). This is a missense mutation, p.P584L, which alters a highly conserved amino acid in the juxtamembrane region of the protein (Figure 1). The change was predicted to be “probably damaging” using Polyphen2 with a score of 0.997 (sensitivity: 0.41; specificity: 0.98) and “not tolerated” using SIFT. Another affected individual from the family, 115-2, also carried the mutation; which was absent in 270 unrelated controls. This is the first mutation to be identified in the juxtamembrane region of the protein. Autophosphorylation of tyrosine residues in this domain of Eph receptors regulates their activity [23]. Tyrosine residues proximal to the altered proline, at positions 588 and 594 in human EPHA2, are phosphorylated [24]. Targeted mutation of these tyrosine residues in murine EPHA2 affects receptor interaction with downstream signalling molecules [25]. Therefore the identified missense mutation may modify downstream EPHA2 signalling during lens development and lead to congenital cataract in Family 115.

**Figure 2:**
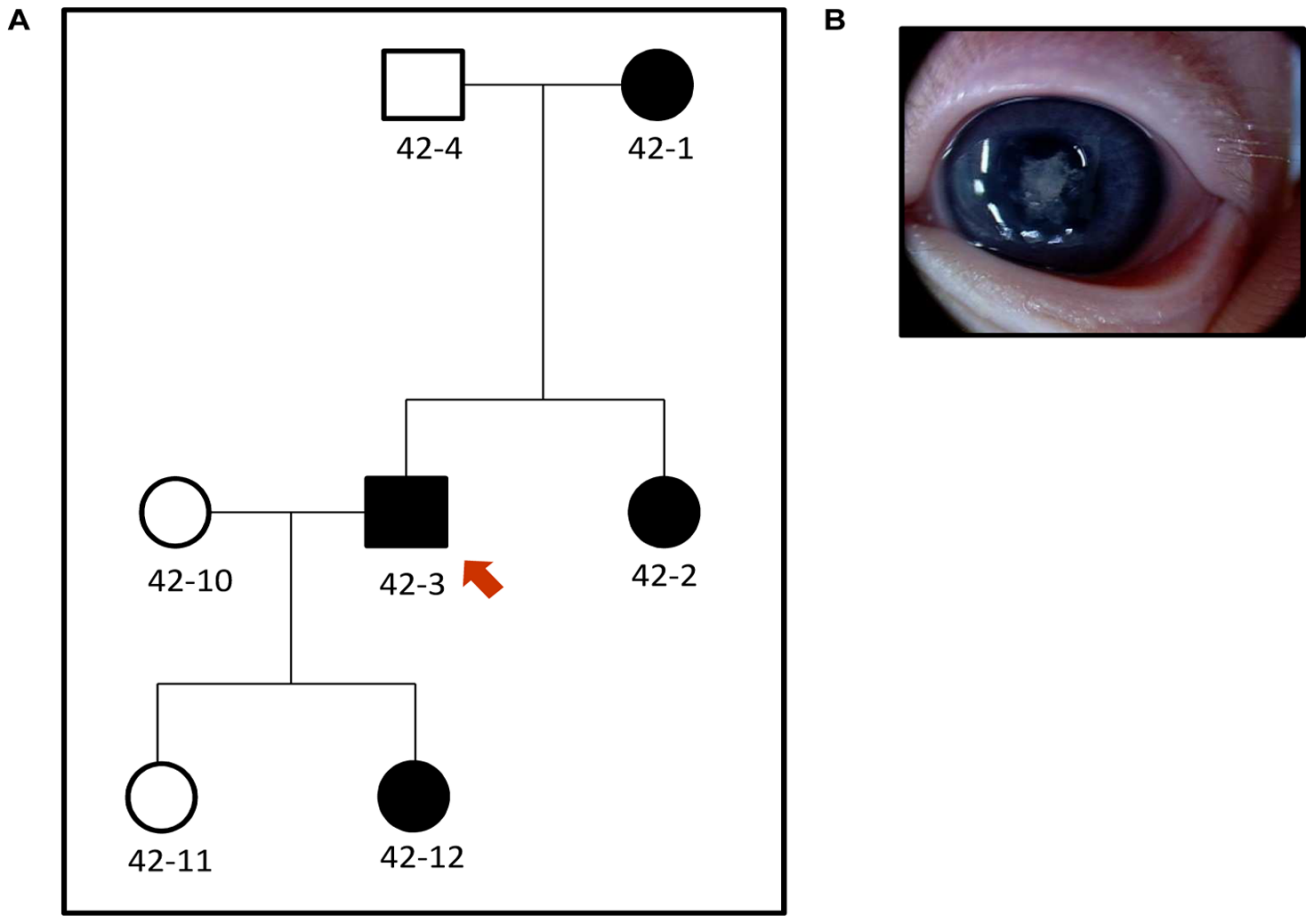
Congenital cataract in Family 42. a) Pedigree of the Family 42. Solid boxes and circles indicate affected individuals. The individuals from whom DNA was available have been given a number. The proband is marked with a red arrow. b) Cataract phenotype of 42-12 showing nuclear cataract.

**Figure 3:**
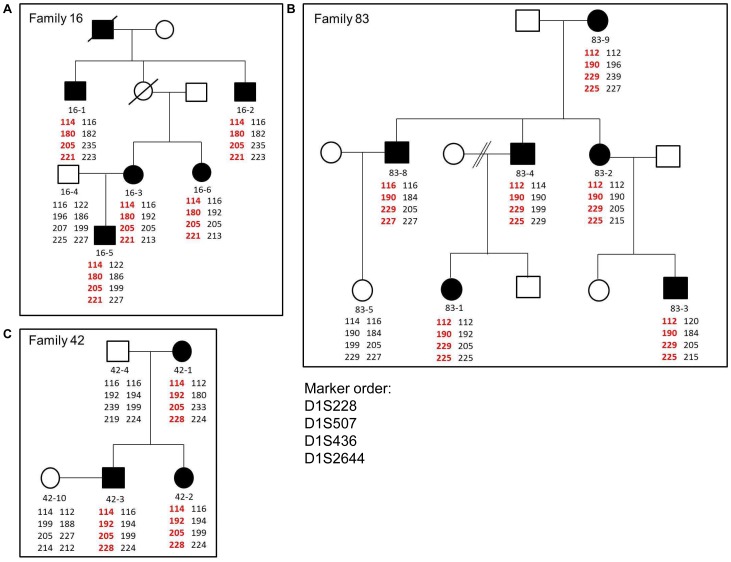
Haplotype analysis in Families 16, 83 and 42. Pedigrees of the Families a) 16, b) 83 and c) 42. Solid boxes and circles represent affected family members. The individuals from whom DNA was available were given a number. Family 83 and 42, from the present study, carry the same splice mutation c.2826-9G>A as was previously reported in Family 16. Haplotypes of members of each family at microsatellite markers D1S228, D1S507, D1S436 and D1S2644 are shown. Haplotype carrying the diseased-allele is represented in red and the normal allele in black. The haplotype carrying the segregating disease allele is different in the three families.

In Family 42, proband 42-3 presented with bilateral nuclear lens opacities and 42-12 was subsequently diagnosed with the same phenotype (Figure 2). We found a splice mutation, c.2826-9G>A in Intron 16 in the proband, which segregated with the disease in the family. The same mutation was found in the proband from Family 83, 83-9, and also segregated with the disease in this family. Although we do not have any information about the cataract phenotype of the proband from Family 83, two other affected family members, 83-1 and 83-3, presented with severe bilateral nuclear cataracts. The splice mutation found in both the families, leads to an insertion of 7 bp in the transcript that in turn leads to a frame-shift and addition of 71 aberrant amino acids in the SAM domain of the translated protein [6]. This mutation has been previously reported to cause total congenital cataract in an Australian family (Family 16) by our group [6]. Therefore to test if the three families carrying this mutation were related, we performed haplotype analysis using microsatellite markers spanning a distance of approximately 5 Mbp in the region around *EPHA2*. The haplotype carrying the mutant-allele was different in the three families, suggesting that the mutation was an independent event in the three families (Figure 3) or these families are more distantly related than the resolution of the microsatellite markers could detect. Individual 83-8 from Family 83 carries different alleles at centromeric marker D1S228 and telomeric marker D1S2644 than other affected members of the family (Figure 3) but carries the mutation and the segregating alleles at the two markers closest to *EPHA2*. A mutation event at marker D1S228 and a recombination event at D1S2644 could explain these differences in the distal markers in this patient [26]. Our results suggest that this mutation, which leads to destabilization of the protein and reduced migration activity of cells expressing mutant EPHA2 [27], has occurred multiple times in the South-Eastern Australian population.

**Figure 4:**
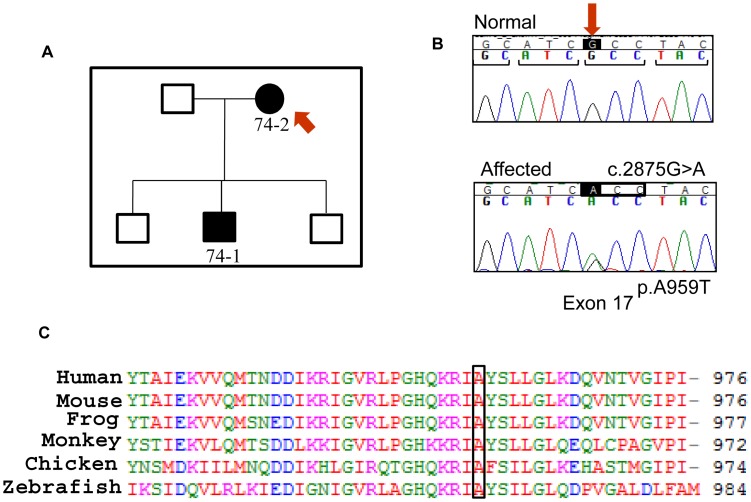
The causative mutation in Family 74. a) Pedigree of the Family 74. Solid boxes and circles indicate affected individuals. The individuals from whom DNA was available have been given a number. The proband is marked with a red arrow. b) Sequence chromatogram showing the variant c.2875G>A, marked by an arrow, present in the proband (bottom). The variant leads to change in a codon, marked by a box, substituting an alanine residue by a threonine in the protein at position 959. The translation reading frame is marked with brackets in the normal sequence (top). c) ClustalW alignment of the region of human EPHA2 including residue 959 showing conservation of the alanine residue across species.

The proband, 74-2, from Family 74 (Figure 4) presented with bilateral subcapsular and cortical opacities at 17 years of age and was found to carry a non-synonymous variant, c.2875G>A in Exon 17 of the gene. This variant leads to a missense change, p.A959T, in the SAM domain of the protein at a residue that is highly conserved across species (Figure 4). Another affected family member, 74-1, presented with bilateral posterior subcapsular cataract at 20 years of age and carries the same variant. Mutations in SAM domain affect EPHA2 downstream signalling [27] and therefore this variant may have a functional effect on the protein. Furthermore, an altered residue at this position can potentially affect the phosphorylation profile of the adjacent phosphorylated tyrosine [25]. Consistent with this, the change was predicted to be “probably damaging” using Polyphen2 with a score of 0.995 (sensitivity: 0.68; specificity: 0.97) and as “not tolerated” using SIFT. Age of diagnosis and cataract phenotype of the affected individuals in family 74 suggest that the variant led to late-onset inherited cataract in the family.

This variant was present in 2 out of 218 unrelated South Australian controls. On further investigation we found that both these individuals had bilateral age-related cataract. One of them had anterior cortical cataract in one eye and congenital with cortical senile cataract in the other eye. This individual had been known to have cataract of a congenital nuclear pattern for many years but did not have cataract surgery until 70 years of age suggesting mild congenital cataract that did not significantly affect vision. The other individual had developed bilateral cortical cataract and undergone surgery at 80 years of age. This DNA change was subsequently listed in dbSNP as rs139787163, following its identification in the Exome Sequencing Project at a frequency of 3 alleles in over 10,000 individuals [22]. Therefore to investigate if the presence of this variant in cataract affected individuals was coincidental, additional BMES age-related cataract cases and controls were screened but none of them carried the variant. Therefore the presence of this rare variant only in cataract affected individuals suggests that it may increase susceptibility to cataract development, although with varied penetrance, severity and age of onset. The previously studied synonymous SNP rs3754334 in Exon 17 of the gene, associated with age-related cataract in Italian and American populations, is directly adjacent to this non-synonymous variant [5], [10]. This suggests that this region of the gene may be important in the mechanism of cataract formation. The spectrum of age of cataract development in carriers of this variant suggests that cataract is perhaps a continuous spectrum of disease and the presence of other genetic and/or environmental factors affects the rate of progression and severity. This variant provides a link between congenital and age-related cataract making it an interesting candidate for further studies.

To date, most of the autosomal dominant congenital cataract causing mutations in the *EPHA2* gene have been reported in the SAM domain of the protein affecting EPHA2 protein function (Table 1) [27]. We report the first autosomal dominant mutation in the juxtamembrane domain of the protein. Interestingly, a mutation in the SAM domain, one in the tyrosine kinase domain and the novel mutation in the juxtamembrane region found in this study, all can lead to nuclear or total cataracts (Table 1) [6], [7]. During lens development, the lens nucleus is formed first by the primary lens fibre cells arising from the posterior lens epithelium, and then secondary lens fibre cells differentiate from the epithelium at the lens equator to form the lens cortex that constitutes the bulk of the lens [28]. Nuclear and total cataract phenotypes suggest a critical role of EPHA2 in primary fibre cell formation. In addition, higher expression of EPHA2 in cortical fibre cells [10], [29] and association of SNPs in the gene with age-related cortical cataract suggest a role of the protein in secondary/cortical fibre cells, later in life. This is also supported by the fact that a rare variant in the gene leads to late-onset cortical or subcapsular cataract in the affected individuals in Family 74. Thus *EPHA2* plays an important role in maintaining lens transparency throughout life.

**Table 1:** Summary of the known congenital cataract causing mutations in the *EPHA2* gene.

DNA change	Protein change	Location in protein domain	Inheritance	Phenotype	Reference
c.1751C>T	p.P584L	Juxtamembrane	Dominant	Nuclear	This study
c.2353G>A	p.A785T	Tyrosine kinase	Recessive	Nuclear	[7]
c.2668C>T	p. R890C	Between tyrosine kinaseand SAM domain	Dominant	Posterior polar	[8]
c.2819C>T	p.T940I	SAM	Dominant	Posterior polar	[6]
c.2826-9G>A	p.D942fs+C71	SAM	Dominant	Total/nuclear	[6] and this study
c.2842G>T	p.G948W	SAM	Dominant	Posterior polar	[5]
c.2875G>A	p.A959T	SAM	Dominant	Subcapsular and Cortical	This study
c.2915_2916delTG	p.V972GfsX39	SAM	Dominant	Posterior polar	[6]

In conclusion, this study suggests that mutations in the *EPHA2* gene account for approximately 4.7% of inherited cataract cases in South-Eastern Australia. The frequency of mutations in five crystallin genes in a subset of this Australian cohort was reported to be 5.2% [30]. The current study indicates that the frequency of congenital cataract causing mutations in the *EPHA2* gene alone is comparable to that in the whole group of crystallin genes taken together. This suggests that mutations in other structurally and functionally important genes such as *EPHA2* in the lens may contribute a significant proportion of the congenital cataract burden.

## Supporting Information

Table S1 Primer sequences for PCR amplification of coding regions of *EPHA2* used for in-house sequencing.
(DOCX)

Table S2 Primer sequences for PCR amplification of coding regions and 3′UTR of *EPHA2* used at AGRF.
(DOCX)

Table S3 Primer sequences for PCR amplification of microsatellite markers around *EPHA2*.
(DOCX)
