# Supplementary material for: Mutations in the EPHA2 Gene Are a Major Contributor to Inherited Cataracts in South-Eastern Australia
Source: PLoS One. 2013 Aug 27;8(8):e72518. doi: 10.1371/journal.pone.0072518 (PMC3754966; doi:10.1371/journal.pone.0072518)
Supplement: Table S1 — Primer sequences for PCR amplification of coding regions of EPHA2 used for in-house sequencing. (DOCX) [file pone.0072518.s001.docx]

**Table S1: Primer sequences for PCR amplification of coding regions of *EPHA2* used for in-house sequencing.**

| **Exon** | **Forward Primer** | **Reverse Primer** |
| --- | --- | --- |
| 1 | GGCCCCTTTAAAGACATTCC | CGACACCAGGTAGGTTCCAA |
| 2 | CTGGAGGGATCCTCACCTTT | GTGAGAAGCTGGACCCTGAG |
| 3 | F1- GGAGAGCACGAACTGGAAAG  F2- GATGAGATCACCGTCAGCAG | R1- AGGCCAGGTAGAAGCCTTTG  R2- AGCAGGGATGAGCTTACCAA |
| 4-5 | GGGGTGGAAGCAGATTGAA | GTCCTCCTTAAGCCCCACCT |
| 6 | GGCTCCACGTCCACTTGT | TCAGATGGCTGGGTGGTT |
| 7 | ATTCCGAGCCTCAGTTTCCT | TCCTTTCCCAAGATGTCTCAA |
| 8 | CTCTGGAGCCTTCCCAAGT | TGAGGAAATGGAGGTTCCTG |
| 9 | ACTGGGCCGCATTCTGAG | CAGTGCCCTGGGAACACC |
| 10-11 | TGACCTTCTCCTCTGACTCCA | GTGGGCACAGTCACAGACAG |
| 12-13 | ACCTTCCCCCATATCTGTGC | AGGTGTGCAGGTGAGAGGAC |
| 14 | CTTGGCTGCAATGGTCCT | AGAGCAAAACTCCGTCTCCA |
| 15 | TTCTGGGATGTTCCTGTTCC | GTGGCCACTCTACCGAAGTG |
| 16 | TCATCCAGGTTAGGGAGCAG | GTTCTGCCCTTCTCTTCCAA |
| 17 | CCAGAGCTCTCTTGCCCTAC | GCAGGGGGAGGAAAGAACTA |
