# Supplementary material for: Mutations in the EPHA2 Gene Are a Major Contributor to Inherited Cataracts in South-Eastern Australia
Source: PLoS One. 2013 Aug 27;8(8):e72518. doi: 10.1371/journal.pone.0072518 (PMC3754966; doi:10.1371/journal.pone.0072518)
Supplement: Table S2 — Primer sequences for PCR amplification of coding regions and 3′UTR of EPHA2 used at AGRF. (DOCX) [file pone.0072518.s002.docx]

**Table S2: Primer sequences for PCR amplification of coding regions and 3’UTR of *EPHA2* used at AGRF.**

| **Exon** | **Forward Primer** | **Reverse Primer** |
| --- | --- | --- |
| 1 | AGGGCATGAATGAACAGGAG | CTGAAACCGCTTATTCTCCG |
| 2 | TGTCTGAGAGTTGGGGTTCC | GCCAGAGATCCCTCTGACTG |
| 3 | F1- TAGTGCCCTACTCTGCCACC  F2- ACTACGGCACCAACTTCCAG | R1- GGCACCGATATCCTGGAAG  R2- AACCTGGGAATGCAGAACC |
| 4 | CTTCTTGCAGGAGCCAGTG | GAGGAGTCAGTGCTGTGCTG |
| 5 | CAGCACAGCACTGACTCCTC | TCTCTAAACACACCCGAGCC |
| 6 | CTGTGGAGGATGTGGCTCC | GCCATGTCTTCTCTCGTACAAATC |
| 7 | GCTAGCTTGGGGTGGTCTC | CACACCACTGTCGTGAATCC |
| 8 | GGAACTCCCTTGTCATCAGC | CGAAGGCATTCCCATTAGAG |
| 9 | AGACCTCACTGACCTCCCTG | CGAGTGACACAGGATGGATG |
| 10 | TCCTCTGACTCCAGAGCACC | AAGTCCACTCGCTGCTTCTC |
| 11 | CACATATGAGGACCCCAACC | GACAGAGCCCCTGCTAAGTG |
| 12 | TCAATACCTGTGCCCTCCTC | CTGAACTCGCCATCCTTCTC |
| 13 | TTCCTTCGGGTAAGGATGTG | AGGTGTTCTGCCTCCTGAAG |
| 14 | ACCACCAGTGTAAGTTGGGG | TAAACTGTCCTCTGCCCAGC |
| 15 | AGGCCCTTCCTGTCTGTTTC | GTGGCCACTCTACCGAAGTG |
| 16 | TGAACCCTACTTGACTGGGC | TCTTCCAAGGAGCCTCTTCC |
| 17 | CTCTTGCCCTACAGGTCCC | TGCTAAGTGCTCAGCTGTGTG |
| 3’UTR | F2- TAGTTCTTCCTCCCCCTGC  F3- ACTGGGTGAGACCCAAAGC  F4- CCCATCTCTCATCCTTTTGG | R2- ACCTCAACACAACCAAGCATC  R3- TCCCAAGACTGAAAGCCAAG  R4- TTGCTGTTCTCCTACCTCCC |
