# Supplementary material for: Mutations in the EPHA2 Gene Are a Major Contributor to Inherited Cataracts in South-Eastern Australia
Source: PLoS One. 2013 Aug 27;8(8):e72518. doi: 10.1371/journal.pone.0072518 (PMC3754966; doi:10.1371/journal.pone.0072518)
Supplement: Table S3 — Primer sequences for PCR amplification of microsatellite markers around EPHA2. (DOCX) [file pone.0072518.s003.docx]

**Table S3: Primer sequences for PCR amplification of microsatellite markers around *EPHA2*.**

| **Marker** | **Forward Primer** | **Reverse Primer** |
| --- | --- | --- |
| D1S228 | FAM-AACTGCAACATTGAAATGGC | GGGACCATAGTTCTTGGTGA |
| D1S507 | FAM-AGGGGATCTTGGCACTTGG | CTCTAGGGTTTCTGGAAAATGCTG |
| D1S436 | HEX-TGAATGTGTCTCCAGTGTTAGC | CTGTAGAGCAATCTGGCAATATGT |
| D1S2644 | FAM-TGCAACCCACCTGAATGA | TACGTGAAGTGCCAGCACA |
